# Semiconductor to metallic transition under induced pressure in Cs_2_AgBiBr_6_ double halide perovskite: a theoretical DFT study for photovoltaic and optoelectronic applications

**DOI:** 10.1039/d1ra03161a

**Published:** 2021-07-07

**Authors:** Md. Nurul Islam, Jiban Podder, Tusar Saha, Protima Rani

**Affiliations:** Department of Physics, Bangladesh University of Engineering and Technology Dhaka-1000 Bangladesh jpodder59@gmail.com

## Abstract

Inorganic double halide perovskites have a wide range of applications in low-cost photovoltaic and optoelectronic devices. In this manuscript, we have studied their structural, electronic, mechanical and optical properties using density functional theory (DFT) simulations. In this work, hydrostatic pressure is induced from 0 to 50 GPa. Disordered Ag and Bi atoms have a large impact on band gap energy; in this case, the indirect band gap is transferred towards a direct band gap. We have seen that pressure-driven samples have transformed a band energy semiconductor into a metallic one. Under the induced hydrostatic pressure, the covalent bond is transformed into a metallic bond and the bond lengths are reduced. Meanwhile, pressure-induced samples enhance symmetry breaking in [AgBr_6_]^5−^ and [BiBr_6_]^3−^ octahedra, which reduces the density of states of the Fermi surface and lowers the total energy. The mechanical behaviors demonstrated that the studied materials are mechanically stable as well as ductile and their ductile nature is enhanced by the driving pressure. The absorption peak is shifted towards the low energy region with increased hydrostatic pressure. The absorptivity and dielectric constant values are also increased with driving pressure. Phase transformed double halide perovskites triggered by outside stimuli produce several outstanding materials properties, giving great scope for a broad range of applications. This type of pristine and disordered double halide perovskite with pressure-driven semiconductor-to-metal phase transition samples may have potential applications in optoelectronic and photovoltaic devices.

## Abstract 2

Inorganic double halide perovskites have a wide range of applications in low-cost photovoltaic and optoelectronic devices.

## Graphical abstract

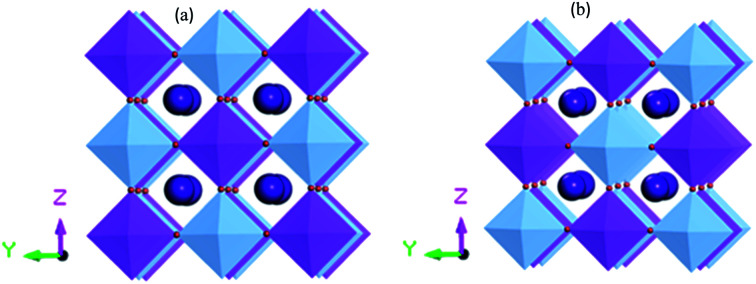

## 1. Introduction

In recent years, lead-free double halide perovskites have been considered promising candidates for versatile applications in low-cost photovoltaic and optoelectronic devices because of their unique electronic and optical properties.^1–5^ Practical applications of inorganic halide perovskites have increased to a large scale, such as a light emitting diodes (LEDs), lasers, radiation detectors, and solar cells. Pb-based hybrid halide perovskites have superior and exceptional photovoltaic properties due to their suitable direct band gap, high absorption coefficients, effective masses of valence electrons and holes, defect tolerance, and carrier diffusion length.^6–8^ In spite of breakthroughs, Pb based hybrid halides will have no use in the long term, because of their toxic impact on the environment.^9^ There is a great challenge for a materials scientist to find out the stable nature of a non-toxic double metal halide for low-cost optoelectronic device applications beyond all these restrictions. The well-known chemical formula of a double metal halide is A_2_M^+^M^3+^X_6_, where A is CH_3_NH_3_^+^ or Cs^+^, M^+^ (Na^+^, Cu^+^ or Ag^+^) is a monovalent cation, M^3+^ (Bi^3+^, Sb^3+^ or In^3+^) is a trivalent cation, and X (Cl^−^, Br^−^ or I^−^) is a halide. In recent years, a new class of double halide perovskite has led to a new generation that provides potential applications in optoelectronic devices.^10,11^ Bi-based organic and inorganic double halides are used in solar cell devices due to the ion's migration easily occurring within monovalent and trivalent cations. Lead free-halide Cs_2_AgBiBr_6_ is an indirect gap semiconductor with a band gap of 1.93 eV, while (Ag, Bi) disorder has a large impact on the band gap energy.^12^ The disordered (Ag, Bi) of Cs_2_AgBiBr_6_ is found to have a direct band gap of nearly 0.44 eV. Herein, we have applied variable pressure on the band structure of Cs_2_AgBiBr_6_, based on first-principles simulations. The Ag 4d-electron orbitals mainly dominate the band gap energy. In the disordered sample, Ag-3d and Bi-6p orbital electrons have undergone hybridization owing to the reduced band gap energy.

The valence band maximum (VBM) and the conduction band minimum (CBM) lie at several *k*-points in the Brillouin zone. This is essential for understanding the pressure-dependent real space charge distribution at different *k*-point energies. Applying a hydrostatic pressure to a material can easily tune the material's various properties.^13–15^ L. Wang *et al.* reported on the influence of lead halide perovskite CH(NH_2_)_2_PbBr_3_ and found that the structural phase is changed at 2.2 GPa.^16^ Pressure-dependent samples undergo band-gap energy shrinkage and the electron orbits move toward the electric field. As a result, the bonding energy is changed within the octahedral state, which mostly affects the boundary conditions of the electronic wave functions and brings about a reduced band gap energy. We have investigated whether the absorption peak is red shifted or blue shifted due to the distortion occurring within [AgBr_6_]^5−^ and [BiBr_6_]^3−^ octahedral states under induced pressure. In particular, the quenched absorption peak of the double halide Cs_2_AgBiBr_6_ was slightly blue-shifted compared to the primary peak under zero pressure conditions.^17^ In this manuscript, we are researching the effects of (Ag, Bi) disorder and pressure induced in Cs_2_AgBiBr_6_ for optoelectronic and photovoltaic applications, applying density functional theory (DFT) *via* investigating the electronic, mechanical and optical properties. A combined characterization implies that Cs_2_BiAgBr_6_ is a potential material for applications in photovoltaic and optoelectronic devices, especially solar cells and photocatalytic activity.

## 2. Theoretical methodology

All the density functional theory (DFT) simulations were performed using plane-wave-based CASTEP code, a module of the studio 8.0 package.^18,19^ The non-spin polarized Perdew–Burke–Ernzerhof (PBE) functional within the general gradient approximation (GGA) method was chosen to describe the exchange–correlation potential and projector augmented-wave (PAW) pseudopotentials.^20–22^ We used the simulations of pristine and disordered Cs_2_AgBiBr_6_ in 4 × 4 × 4 gamma-centered (*Γ*) *k*-points. 5s^2^ 5p^6^ 6s^1^ for Cs, 4d^10^ 5s^1^ for Ag, 6s^2^ 6p^3^ for Bi, and 4s^2^ 4p^5^ for Br were some of the valence band electronic configurations used in these partial density of states (PDOS) calculations. The unit cell structure was constructed into a 1 × 1 × 1 supercell model for all simulations.^23^ In these calculations, the cutoff energy was chosen as 420 eV. A scissor value (0.25 eV), a disparity between the theoretical values (1.91 eV) and the experimental values (2.16 eV) in the band gap of the Cs_2_AgBiBr_6_ were used for the absorption and dielectric function property calculations.^24,25^ The studied sample was entirely optimized by reducing the total energy, internal forces, and external stresses, varying the constant lattice parameters and internal coordinates simultaneously by applying the Broyden–Fletcher–Goldfarb–Shanno (BFGS) algorithm. The unit cell structure and atomic relaxations were accomplished so that the residual forces were below 0.03 eV Å^−1^. Within the CASTEP code, the elastic modulus *C*_*ij*_ is simulated by finite-strain theory^26–28^ and the consequence of external stresses. The stress tensor has six stress parameters *σ*_*ij*_ for each strain *δ*_*j*_ employed on the unit cell. The lattice dynamic properties such as phonon dispersion were calculated by employing the finite displacement supercell approach.

## 3. Results and discussion

### 3.1. Structural aspect and phase stability

**Fig. 1:**
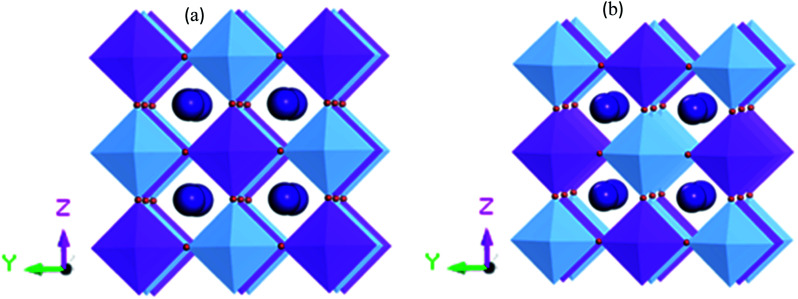
(a) Crystal 1 × 1 × 1 supercell structure for pristine (CASTEP calculation) and (b) disordered Cs_2_AgBiBr_6_ (CASTEP calculation). The cyan, blue, and purple spheres represent Cs, Ag, Bi and Br atoms, respectively.

Fig. 1 shows the supercell structure of pristine and disordered Cs_2_AgBiBr_6_ (CASTEP). Lead-free metal double halide perovskites Cs_2_AgBiBr_6_ has a cubic phase in the space group *Fm*3̄*m* (no. 225). In the unit cell structure, Cs atoms have a face-centered position with an 8*c* Wyckoff site and fractional coordinates (0.25 0.25 0.25), the Bi atoms have a body-centered position with a 4*b* Wyckoff site and fractional coordinates (0.5 0.5 0.5), Ag atoms are located in the corner positions with a 4*a* Wyckoff site and fractional coordinates (0 0 0) and Br atoms have face-centered positions with a 24*e* Wyckoff site and fractional coordinates (0.2513 0 0). In our case, the samples have two octahedral sites AgBr_6_ and BiBr_6_.

We calculated the tolerance factor from the equation *t* = (*R*_A_ + *R*_X_)/√2[(*R*_B_^/^ + *R*_B_^//^)/2 + *R*_X_]. The corresponding range of stable structure is 0.81 < *t* < 1.0. Within this range, the octahedral factor is identified as *μ*= (*R*_B_^/^ + *R*_B_^//^)/2*R*_X_, and the stability of the structure lies in the range 0.81 < *μ* < 1.0.^29^ To obtain the structural stability, we applied the Shannon ionic radius. For Cs_2_BiAgBr_6_ with bromide halide, we calculated (*μ*, *t*) = (0.41, 0.92), which is identified with standard halide perovskites.

**Fig. 2:**
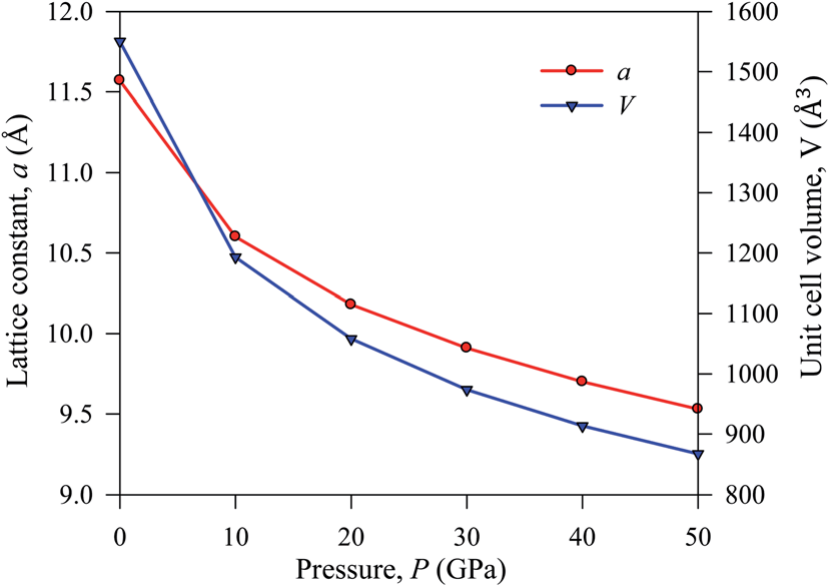
Lattice constant *a* and cell volume *V* of pressure-induced sample Cs_2_AgBiBr_6_.

The simulated lattice parameters *a* and the corresponding unit cell volume *V*_0_ with previously published experimental and theoretical results of Cs_2_AgBiBr_6_ are presented inTable 1. We conducted a DFT simulation driving various hydrostatic pressures from 0 to 50 GPa for Cs_2_AgBiBr_6_. Under ambient pressure, the simulated theoretical lattice parameters in this work are considered a good fit with previously published theoretical work. The DFT-based calculated lattice parameter is slightly higher than the experimental finding, which is a limitation of the GGA approach. The lattice parameter and unit cell volume are changed under driving hydrostatic pressure and are displayed in Fig. 2. From Fig. 2, it is confirmed that the values of lattice parameters and unit cell volumes are decreased by applying various hydrostatic pressures due to the space between lattice vacancies and the bond lengths being reduced. As a result, repulsive phenomena between atoms have become more robust, increasing the difficulty of crystal compression under an applied pressure.

**Table 1:** The calculated and available published experimental and theoretical lattice constant *a* and unit cell volume *V* of Cs_2_AgBiBr_6_ under various pressures

Pressure (Gpa)	*a* (Å)			*V*_0_ (Å^3^)
	Present study	Simulation	Experimental	
0	11.57	11.49 (ref. 31)	11.27 (ref. 30)	1550.32
10	10.60	—	—	1192.96
20	10.18	—	—	1057.99
30	9.91	—	—	973.63
40	9.70	—	—	913.32
50	9.53	—	—	867.08

### 3.2. Electronic properties

**Fig. 3:**
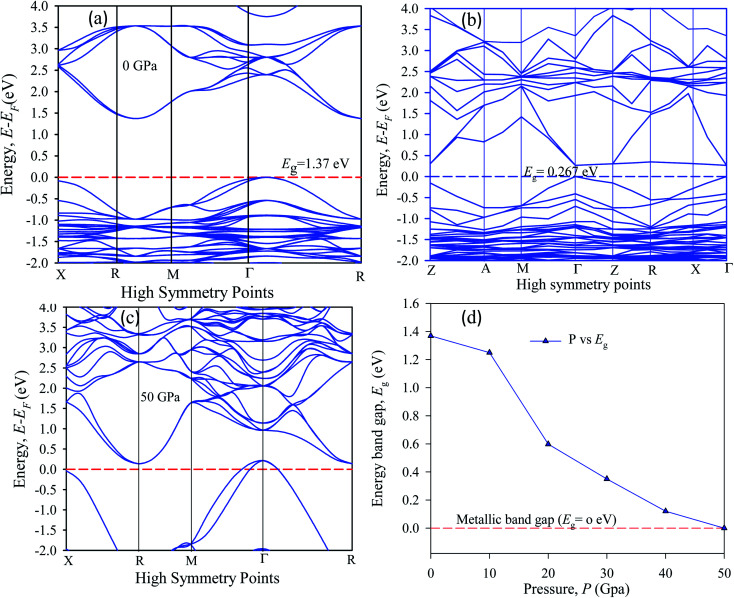
The electronic band structure of (a) pristine, (b) disordered, (c) pressure-induced and (d) pressure-varying energy gap of Cs_2_AgBiBr_6_.

The band energy calculation was carried out at hydrostatic pressure to study pressure-induced band structure variation in Cs_2_AgBiBr_6_. The present band gap energy values are in good agreement with those in other manuscripts.^32–35^ The simulated band structure is shown in Fig. 3. A hybrid potential like HSE (Heyd–Scuseria–Ernzerhof) may be a better estimate for exact band gap measurements. But it does not fit for some samples. Thus, it is still challenging to search for the appropriate potential to predict the theoretical electronic band gap for the estimated samples. However, the main aim of this research is to investigate indirect to direct band gap conversion, and semiconductor to metal phase transformation due to ordered and disordered systems and the band gap limitation is ignored for the GGA approach. We have seen that the bottom of the conduction band (CB) and the top of the valence band (VB) are located at dissimilar (*R* ↔ *Γ*) *k*-points.

In our samples, the calculated electrical band gap energy is lower than the absorption spectra threshold energy, which indicates that the ordered samples have an indirect band gap nature and the opposite is found for disordered samples. The indirect nature semiconductor is an effective candidate for photovoltaic applications. The indirect band sample identifies that the electron cannot move directly from the high energy states in the valence band to the lower energy state in the conduction band, without undergoing any changes in *k*-point energy. These dissimilar *k*-points indicated that the structure of Cs_2_AgBiBr_6_ was an indirect band gap semiconductor and the value of the band gap was decreased under induced pressure. Under induced pressure, there was band-gap shrinkage and orbital movement towards the electric field (EF). For a 50 GPa pressure-induced sample, as presented in Fig. 3, electronic localization began to decrease the band gap across the Fermi level. After an increase in the induced pressure the sample ultimately achieved a metallic band nature. The band structure results confirmed that Ag–Bi disorder has a greater impact on the band gap energy. Fig. 3(b) shows that the maximum conduction band and the minimum valence band are lying at the same *k*-point energy, which indicates that the band structure is a direct semiconductor in nature.

**Fig. 4:**
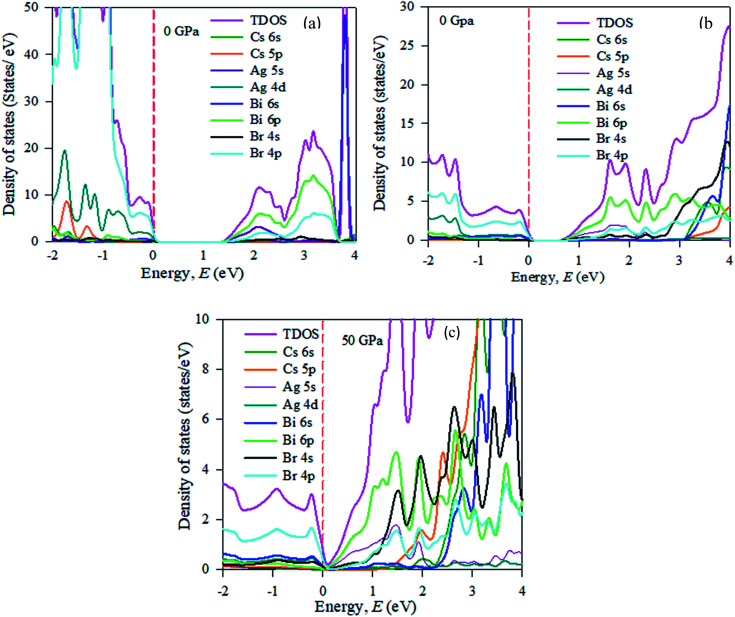
The partial density of states (PDOS) of (a) pristine, (b) disordered, and (c) pressure-induced Cs_2_AgBiBr_6_.

In the band structure, energy is shifted towards the lower energy region owing to Bi and Ag ions creating defect energies of Br 4p and Bi 6s orbitals. Band gap shifting and phase transition samples may be suitable for a broad range of applications. The total density of states (TDOS) and partial density of states (PDOS) of Cs_2_AgBiBr_6_ are presented in Fig. 4. From the TDOS and PDOS in the figure the valence band energy is mainly composed of Ag-4d and Br-6s orbitals with little contribution from Cs-6s and Cs-3p states. The high energy band is mainly attributed to the Ag-4d orbital with a small contribution from Cs-6s and Cs-5p electrons.

The band structures have been influenced by the hydrostatic pressure, and the maximum conduction band (CB) of Cs_2_AgBiBr_6_ has been enhanced downward into the minimum valence band (VB). As shown in Fig. 3(a), the band gap energy of the studied samples decreases from 1.37 eV to zero when the hydrostatic pressure reaches 50 GPa. Lead-free double halide perovskites, Cs_2_AgBiBr_6_, have displayed great potential applications in photocatalytic devices. Herein, the (Ag, Bi) disorder has a large impact on the band gap energy, transforming the indirect band gap towards the direct band gap. Moreover, the indirect band gap energy creates phonon energy in the material due to producing heat energy and finally decreasing the device's suitability for optoelectronic device applications.

**Fig. 5:**
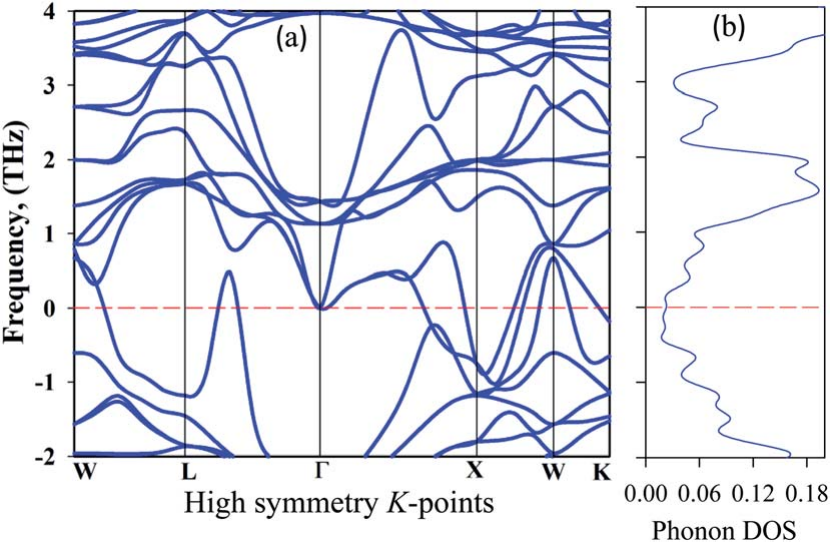
Phonon dispersion curves: (a) high symmetry direction and (b) the phonon density of states of at 50 GPa for Cs_2_AgBiBr_6_.

To be stable, natural gain in a material needs to fulfil some criteria. Firstly, for mechanical stability, a sample must fulfil a set of elastic moduli conditions. For the studied sample, this will be discussed in section 3.3. The second criterion is dynamic stability. For dynamic stability in a sample the crystal lattice must be invariable. The second condition is that there should be no soft phonon modes in the phonon dispersion. This condition implies that soft phonon modes are manifested in a set of atoms moving from a high to a low crystal symmetry structure, which means that the sample has an unstable nature. Soft phonon modes have an imaginary (negative) frequency. In the case of a dynamically stable crystal, all phonon frequencies must have positive values. To see the nature of the stability, we analyzed the phonon dispersion curves. It is clear that an imaginary frequency is found at the *W*, *L*, *K*, and *X* points whose phonon dispersion curves are shown below zero frequency, indicating unstable modes. Consequently, no imaginary frequency is found at the *Γ* point, indicating a stable nature (Fig. 5).

**Fig. 6:**
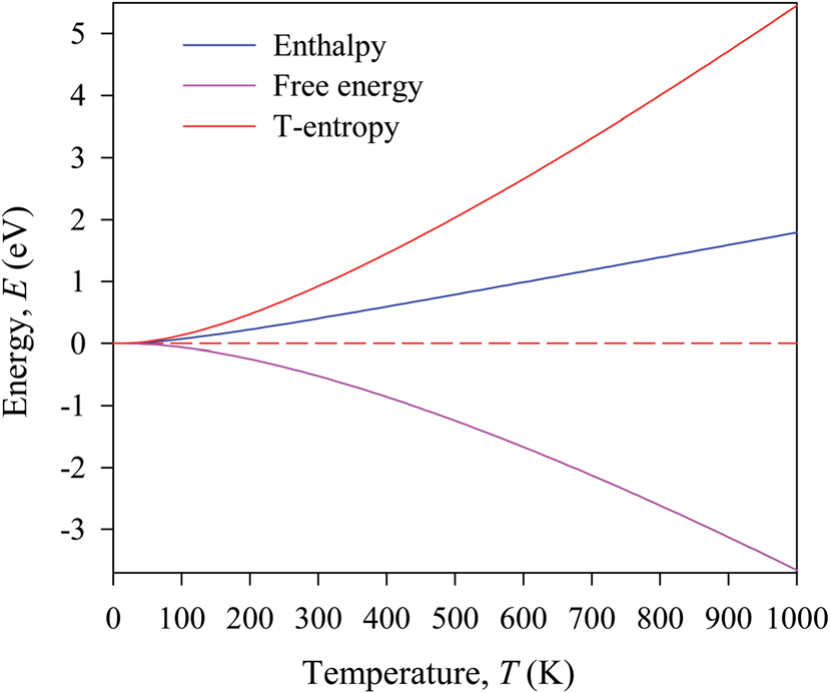
Temperature dependence of the calculated thermodynamic properties of Cs_2_AgBiBr_6_ under a pressure of 50 GPa.

In this manuscript, we have investigated the thermodynamic stability at varying temperatures of the studied sample under 50 GPa pressure. Thermodynamic properties like enthalpy *H*, free energy *F* and entropy *S* at finite temperature were calculated *via* phonon modes. The vibration contribution to the free energy is derived as follows^36^
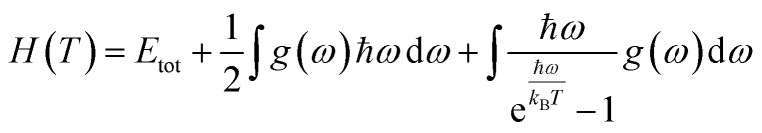

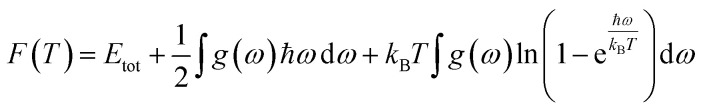

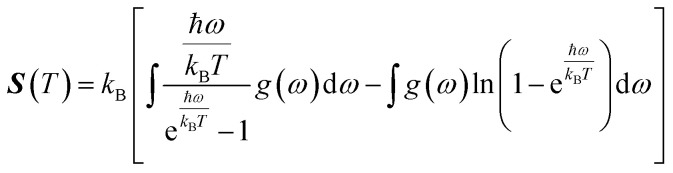
where *g* (*ω*) is the phonon density of states and *k*_B_ is the Boltzmann constant. Fig. 6 shows the temperature-dependent thermodynamic properties. It can be seen from the figure that the enthalpy and free energy increased temperature while the entropy decreases with an increase in temperature. Fig. 6 also shows that when the temperature approaches zero, three terms (*H*, *F* and *S*) approach zero, which agrees with the third law of thermodynamics.

**Fig. 7:**
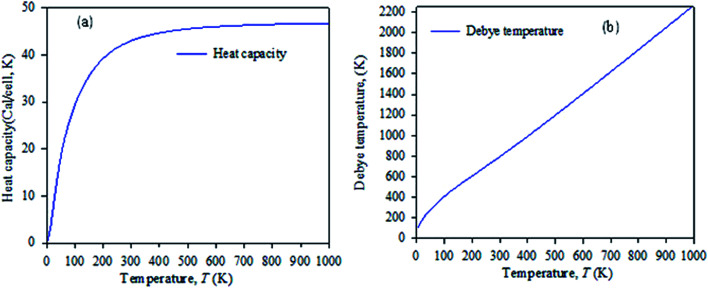
Temperature dependence of (a) heat capacity and (b) Debye temperature derived from the phonon mode.

Fig. 7 shows the temperature dependence of the Debye temperature and heat capacity derived from the phonon mode for Cs_2_AgBiBr_6_ under 50 GPa pressure. It can be seen that heat capacity approaches the Dulong–Petit limit at high temperature. We predicted that our studied samples would have phase stability under 50 GPa pressure.

**Fig. 8:**
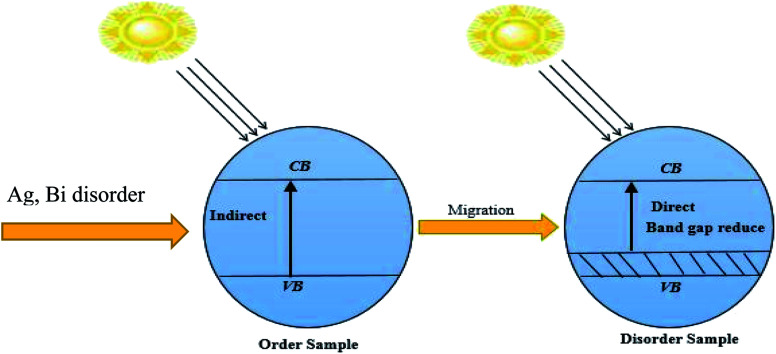
Schematic diagram representing the change in the band gap energy with (Ag, Bi) disorder.

The indirect electrical band gap of pure Cs_2_AgBiBr_6_ shows a longer lifetime of photo-excited electrons and holes than direct electrical band gap semiconductors due to the direct separation of photo-generated electrons from the CB to the VB of a semiconductor not being possible. Fig. 8 represents the photocatalytic activity. The excited electrons from the valance band (VB) are injected into the conduction band (CB), which decreases the gap energy in disordered Cs_2_AgBiBr_6_ lead-free double metal halide perovskite. It shows the impact of the isolation of photo-generated electron–hole pairs, and favors the migration of photoexcited carriers and processing photocatalysis. The disordered sample with new dopant energy levels practically mitigates the band gap energy of the photocatalyst.^37^ The work would be suitable for photocatalytic activity applications.

**Fig. 9:**
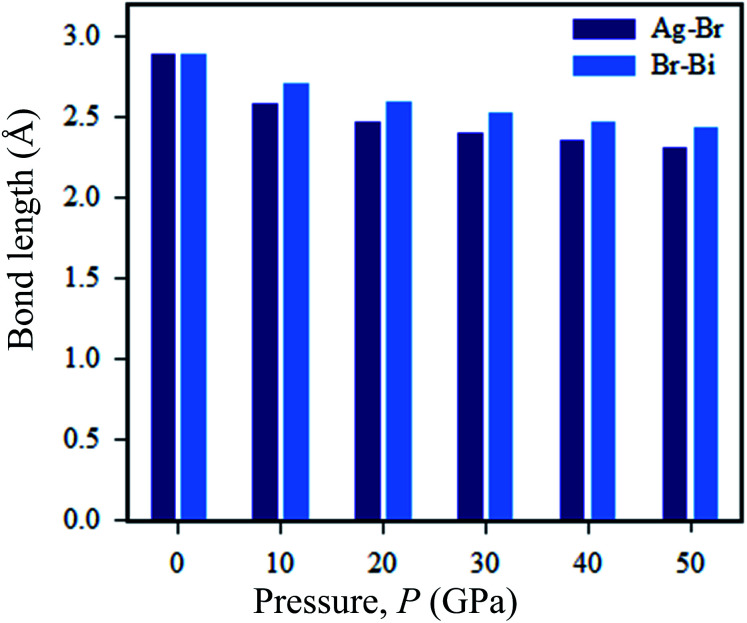
Changes in the Ag–Br and Bi–Br bond lengths under various pressures.

Now we discuss the pressure-driven charge density and Ag–Br and Bi–Br bond length evaluation in Cs_2_AgBiBr_6_. From Fig. 9, we can see that pressure-induced on Ag–Br and Bi–Br, bond lengths decrease with increased driving pressure, due to ionic radii overlapping with each other. Another reason that the inter-octahedral Ag–Br and Bi–Br bond lengths are changed is due to the crystal defects that have occurred with Ag and Bi atoms. The bond length changes with respect to driving pressure, which is easily understood from the viewpoint of the stiffness of the octahedra.

Without pressure, the intra-octahedral Ag–Br and Bi–Br bonds are considerably stronger due to the relatively weak van der Waals forces of the former Ag–Br and Bi–Br bonds. However, under a pressure of 50 GPa the Ag and Bi atoms exhibit weak bonds. Meanwhile, in pressure-induced samples symmetry breaking is occurring in [AgBr_6_]^5−^ and [BiBr_6_]^3−^ octahedra, which is manifested as a reduction in the density of states of the Fermi surface and thus lowers the total energy. The bond length of Ag–Br is decreased when the structure is converted from ordered (2.87 Å) to disordered (2.82 Å) Cs_2_AgBiBr_6_. The tendency of the bond length to shrink in the disordered systems can cause the band gap energy to shift from indirect to direct. In the case of pressure-driven samples, the Ag–Br bond length is decreased with respect to induced pressure, and finally, the samples are converted from semiconductor to metal.

**Fig. 10:**
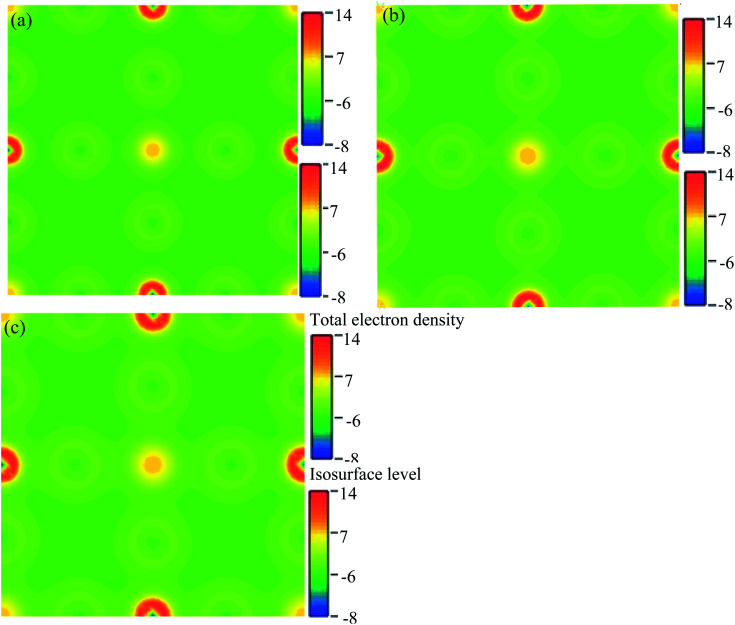
The electron charge density: (a) pristine, (b) disordered, and (c) pressure-induced Cs_2_AgBiBr_6_.

We have researched the electronic charge density (e Å^−3^) distribution in our sample. In the studied samples, spherical-shaped charge densities overlap with each other. Fig. 10 represents the electronic charge density of Cs_2_AgBiBr_6_. The color map on the right-hand side of Fig. 10 shows the total charge density. The charge density separation map identifies that a covalent bond is present in the pure sample. The Bi and Ag atoms have formed a covalent bond at the site with the maximum charge density that exhibits strong electron localization. Under pressure, the bonding charge densities have increased because of the decreasing interatomic distances. As a result of the electric charge, aggregation is increased by driving pressure.

### 3.3. Mechanical properties

The elastic tensor properties are essential parameters for understanding the mechanical nature of crystal-solids. Cubic structure crystals like pressure-induced Cs_2_AgBiBr_6_ have three independent elastic moduli *C*_*ij*_; these are *C*_11_, *C*_12_, and *C*_44_. The simulated elastic parameters are listed in Table 2. The mechanical stability of a crystal can be satisfied with its elastic constants using Born criteria. For a cubic system, the estimated compound, to be mechanically stable, should satisfy the conditions:^34,35^*C*_11_ + 2*C*_12_ > 0, *C*_44_ > 0, *C*_11_ − *C*_44_ > 0 for high symmetry. Additionally, the cubic crystal stability condition: *C*_12_ < *B* < *C*_11_ is also fulfilled by the title compound. Elastically isotropic cubic crystals should satisfy the conditions, 2*C*_44_ = *C*_11_ − *C*_12_. The elastic moduli *C*_12_ and *C*_44_ are different quantities and (*C*_12_ − *C*_44_) is denoted the Cauchy pressure^36,38^ and is distributed as an elementary instrument for computing many phenomena in crystalline solids. In solid samples, Cauchy pressure values with positive and negative signs indicate a metallic or covalent bond nature, respectively. Our studied samples have the positive Cauchy pressure that indicates that the studied sample shows ductile behavior. Moreover, some special mechanical characteristics of the title sample can be observed in Table 3. The Young's modulus value *E* determines the resistance in the opposite direction to the longitudinal tension. It can be seen from Table 3 that the elastic constants increase with an increase in the pressure up to 50 GPa; as a result, the bulk modulus increases.

**Table 2:** Calculated elastic constants *C*_*ij*_ (in GPa) and Cauchy pressure of Cs_2_AgBiBr_6_ under various pressures

Pressure	*C*_11_	*C*_12_	*C*_44_	*C*_12_ − *C*_44_	Ref.
0	59.02	13.37	8.15	5.22	39
0	38.74	7.58	7.46	0.12	This work
10	48.28	16.58	8.20	8.83	This work
20	205.31	58.65	12.53	46.10	This work
30	264.27	71.94	8.44	63.50	This work
40	344.51	96.05	13.95	82.10	This work
50	411.65	114.32	14.46	99.86	This work

**Table 3:** The evaluated mechanical parameters of Cs_2_AgBiBr_6_ under various pressures

Pressure	*B* (GPa)	*G* (GPa)	*Y* (GPa)	*B*/*G*	*v*	Ref.
0	28.58	8.85	—	3.22	0.18	39
0	17.89	10.81	36.35	1.64	0.24	This work
10	27.14	11.26	29.68	2.41	0.31	This work
20	107.54	36.86	99.25	2.92	0.34	This work
30	136.05	43.53	118.00	3.13	0.35	This work
40	178.87	58.06	157.17	3.08	0.35	This work
50	213.43	68.14	184.77	3.13	0.35	This work

**Fig. 11:**
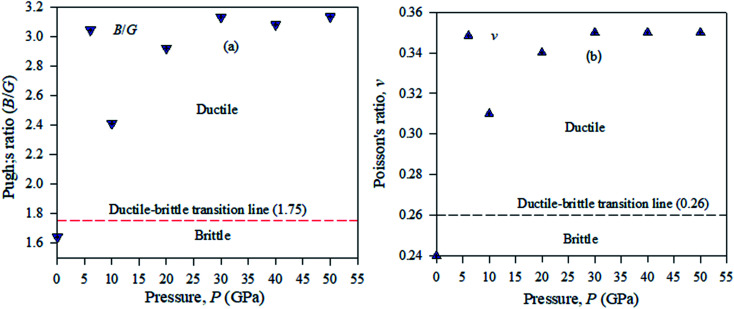
Ductile–brittle nature (a) Pugh's ratio and (b) Poisson's ratio of pressure-induced Cs_2_AgBiBr_6_ samples.

Pugh used the bulk to shear modulus value ratio (*B*/*G*) (brittle/ductile) to identify defects in crystal solids.^39,40^ According to this idea, ductile materials have a *B*/*G* ratio higher than the critical value of 2.46, which is distributed as a broad line between the brittle and ductile natures in crystalline solid samples. A material will exhibit a ductile nature, if its Pugh's ratio has a value greater than the broader line value shown in Fig. 11. To compute Pugh's ratio (ductile/brittle), the failure mode in crystal solids and Poisson's value *v* are applied fruitfully for engineering purposes. In a crystalline solid sample, ductile or brittle natures are identified with a critical range of *v* = 0.32.^41^ It is shown from Table 4 that Cs_2_AgBiBr_6_ is ductile and by employing a pressure up to 50 GPa, this ductility enhances Cs_2_AgBiBr_6_ as a potential component for device fabrication. For the cubic structured Cs_2_AgBiBr_6_, the maximum and minimum values of *Y*, *G*, and *v* are analyzed with the help of the ELATE suit program.^42^ The maximum and minimum *Y*, *G*, and *v* values showed in another way that the sample is isotropic in nature; the crystal is anisotropic in nature. The movement of the spherical shape identifies the rate of elastic anisotropy level of the solids. Table 4 shows the minimum and maximum values of *Y*, *G* and *v* for the pressure-induced Cs_2_AgBiBr_6_ compounds. A high ratio of AE = *E*_Max_/*E*_Min_ indicates significant elastic anisotropy in a sample. The calculated elastic anisotropy values of the studied samples are 1.82 (0 GPa), 1.77 (10 GPa), 4.91 (20 GPa), 9.41 (30 GPa), 7.41 (40 GPa) and 8.53 (50 GPa), for pure and pressure-induced samples of Cs_2_AgBiBr_6_. Their elastic anisotropic nature shows that pressure-induced samples may be perfect for applications by the scientific community.

**Table 4:** The minimum and maximum limits of Young's modulus (*Y*), shear modulus (*G*), and Poisson's ratio (*v*) for pressure-induced Cs_2_AgBiBr_6_

Pressure	Young's modulus per GPa		Shear modulus per GPa		Poisson's ratio	
	*Y*_max._	*Y*_min._	*G*_max._	*G*_min._	*v*_max._	*v*_min._
0	36.35	19.93	15.65	7.58	0.48	0.09
10	39.80	22.36	15.84	8.20	0.53	0.16
20	179.25	36.25	73.33	12.53	0.80	0.05
30	233.48	24.80	96.16	8.44	0.89	0.02
40	302.63	40.79	124.23	13.95	0.86	0.03
50	361.96	42.43	148.67	14.46	0.88	0.03

### 3.4. Optical properties

**Fig. 12:**
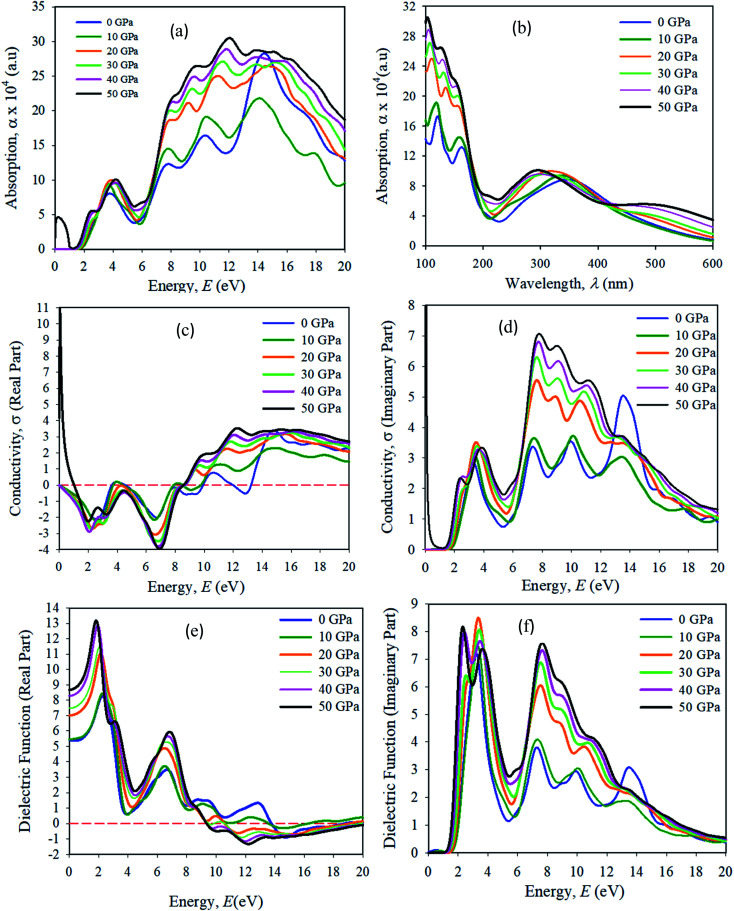
The simulated optical properties (a) absorbance *vs.* energy, (b) absorbance *vs.* wavelength, (c) conductivity, real part (d) conductivity, imaginary part, (e) real dielectric function, (f) imaginary dielectric function of the pressure-induced Cs_2_AgBiBr_6_ samples.

Optical properties, like the absorption spectrum, the real and imaginary parts of optical conductivity, and the dielectric function are shown in Fig. 12. In all of the simulations in this manuscript, a Gaussian smearing of 0.5 eV is used. The optical properties simulation results were taken in the {100} plane orientation. A scissor value is used of 0.25 eV for all optical property simulations. The energy range used was 0–20 eV. The absorption spectra were taken in the UV-vis, and visible wavelength (*λ*) range 100–600 nm. The optical absorption *α*(*ω*) determines the entrance of light at wavelength (*λ*) into a solid sample.^43^ The first absorption peak in the energy range of approximately 3.95 eV is more important for device applications. We have seen that Cs_2_AgBiBr_6_ has a strong absorption spectrum lying in the visible wavelength (nm) area. The first and second absorption peaks are present in the ranges of approximately at 150 and 180 nm, respectively. The optical absorption intensities are apparently increased due to their large band gaps compared to pristine and pressure-induced samples. The absorption spectra output is blue-shifted, and a strong absorption edge is situated at nearly 150 nm. For pressure-induced samples, the key absorption edge is red-shifted and it develops in a lower energy region and increases in intensity. The light absorption spectra of perovskite materials are strongly dependent on the electronic structures. A stronger optical absorption implies an improved photovoltaic performance. Hence the band gap energy was transferred towards the visible region and the maximum absorption peak occurred in the UV-region, which indicates that the studied samples are potential candidates for the optoelectronics industry.

The optical conductivity (1/*f*_s_) is a fundamental parameter for identifying the electromagnetic response of a material.^44^ In another explanation, the optical conductivity implies the amount of photons passed through the samples. It exposes the electrical conductivity when a sample is placed in a strong electric field and it connects the current density to the electric field for natural frequencies. The optical conductivity and electrical conductivity improve with rising photon absorption. It can be seen that the real part vanishes at approximately 12.5 eV, indicating that the sample has an optically anisotropic nature. The optical conductivity has similar features to absorption spectra, as presented in Fig. 12(a), owing to the escape of free carriers from the balance band to the conduction band when it absorbs energy. The amount of electromagnetic radiation response in a sample needs to be understood from the complex dielectric function.^45^

The imaginary part of the dielectric function (*ε*_2_) corresponds to electron excitation. The first peak of the imaginary part of the dielectric function (*ε*_2_) occurs at <1.5 eV due to the intra-band transitions within the Bi 6p and Ag 3d orbital bands. In the spectrum the most essential quantity is the zero-frequency limit *ε*_1_(0), which is the electronic portion of the static dielectric constant. From the real part of the dielectric constant, it is clear that *ε*_1_(*ω*) increases with induced pressure. The *ε*_1_(0) for a pressure-induced sample starts rising from a zero frequency, reaches its maximum peak, then starts to reduce, and in given energy ranges it drops below zero. In these areas, the incident photon beam is totally attenuated.^46^ A combined study of the optical properties of pressure-induced and disordered materials is suitable for optoelectronic and photovoltaic device applications.

## 4. Conclusions

In conclusion, we have applied DFT to calculate the phase stability, and electronic, mechanical, and optical properties of Cs_2_AgBiBr_6_ double halide perovskites. The simulated structural parameters *a* and *V* decreased with an increase in hydrostatic pressure in pristine Cs_2_AgBiBr_6_ samples due to the space of lattice vacancies being reduced. The indirect band energy is transferred to the direct band energy in the case of the structure being converted from ordered to disordered. We have also seen that pressure-driven samples have transformed from semiconductor phase to metallic behavior. With pressure, the Ag–Br and Bi–Br bond lengths are changed due to the crystal defects occurring with Ag and Bi atoms. Meanwhile, in hydrostatic-pressure driven samples, symmetry breaking is occurring in [AgBr_6_]^5−^ and [BiBr_6_]^3−^ octahedra due to a reduction in the density of states and band gap energy. The charge density map confirmed that the covalent bond is present in the pure sample. With increasing pressure, the covalent bond is converted into a metallic bond. The mechanical behaviors demonstrated that perovskite double halide compounds are mechanically stable. Their ductile nature is enhanced with an increase in driving pressure. The elastic anisotropy behaviors showed that pressure-driven samples will be suitable for applications by the scientific community. For the optical properties, we saw that the absorptivity and dielectric constant values also increased with an increase in driving pressure. Phase transferred double halide perovskite materials have provided great scope for a broad range of applications. A combined study suggests that the pressure-induced samples are suitable for optoelectronic devices, especially solar and photovoltaic applications.

## Author contributions

Md. Nurul Islam: conceptualization, data curation, investigation, methodology, formal analysis, original draft writing, Jiban Podder: supervision, formal analysis, review and editing, Tusar Saha: software, formal analysis, and Protima Roy: software, formal analysis.

## Conflicts of interest

There are no conflicts to declare.

## Supplementary Material

## References

[cit1] Zhou H., Chen Q., Li G., Luo S., Song T. B., Duan H. S., Hong Z., You J., Liu Y., Yang Y. (2014). Interface engineering of highly efficient perovskite solar cells. Science.

[cit2] Burschka J., Pellet N., Moon S. J., Humphry-Baker R., Gao P., Nazeeruddin M. K., Grätzel M. (2013). Sequential deposition as a route to high-performance perovskite-sensitized solar cells. Nature.

[cit3] Wong A. B., Lai M., Eaton S. W., Yu Y., Lin E., Dou L., Fu A., Yang P. (2015). Growth and anion exchange conversion of CH_3_NH_3_PbX_3_ nanorod arrays for light-emitting diodes. Nano Lett..

[cit4] Zheng E., Yuh B., Tosado G. A., Yu Q. (2017). Solution-processed visible-blind UV-A photodetectors based on CH_3_NH_3_PbCl_3_ perovskite thin films. J. Mater. Chem. C.

[cit5] Eames C., Frost J. M., Barnes P. R., O’regan B. C., Walsh A., Islam M. S. (2015). Ionic transport in hybrid lead iodide perovskite solar cells. Nat. Commun..

[cit6] Xing G., Mathews N., Sun S., Lim S. S., Lam Y. M., Grätzel M., Mhaisalkar S., Sum T. C. (2013). Long-range balanced electron-and hole-transport lengths in organic-inorganic CH_3_NH_3_PbI_3_. Science.

[cit7] Giorgi G., Fujisawa J. I., Segawa H., Yamashita K. (2013). Small photocarrier effective masses featuring ambipolar transport in methylammonium lead iodide perovskite: a density functional analysis. J. Phys. Chem. Lett..

[cit8] Frohna K., Deshpande T., Harter J., Peng W., Barker B. A., Neaton J. B., Louie S. G., Bakr O. M., Hsieh D., Bernardi M. (2018). Inversion symmetry and bulk Rashba effect in methylammonium lead iodide perovskite single crystals. Nat. Commun..

[cit9] Manser J. S., Saidaminov M. I., Christians J. A., Bakr O. M., Kamat P. V. (2016). Making and Breaking of Lead Halide Perovskites. Acc. Chem. Res..

[cit10] Shi W., Cai T., Wang Z., Chen O. (2020). The effects of monovalent metal cations on the crystal and electronic structures of Cs_2_MBiCl_6_ (M = Ag, Cu, Na, K, Rb, and Cs) perovskites. J. Chem. Phys..

[cit11] Dave K., Fang M. H., Bao Z., Fu H. T., Liu R. S. (2020). Recent Developments in Lead-Free Double Perovskites: Structure, Doping, and Applications. Chem.–Asian J..

[cit12] Yang J., Zhang P., Wei S. H. (2017). Band Structure Engineering of Cs_2_AgBiBr_6_ Perovskite through Order–Disordered Transition: A First-Principal Study. J. Phys. Chem. Lett..

[cit13] Liang Y., Huang X., Huang Y., Wang X., Li F., Wang Y., Tian F., Liu B., Shen Z. X., Cui T. (2019). New metallic ordered phase of perovskite CsPbI_3_ under pressure. Adv. Sci..

[cit14] Yin T., Liu B., Yan J., Fang Y., Chen M., Chong W. K., Jiang S., Kuo J. L., Fang J., Liang P., Wei S. (2018). Pressure-engineered structural and optical
properties of two-dimensional (C_4_H_9_NH_3_)_2_PbI_4_ perovskite exfoliated nm-thin flakes. J. Am. Chem. Soc..

[cit15] Ma Z., Liu Z., Lu S., Wang L., Feng X., Yang D., Wang K., Xiao G., Zhang L., Redfern S. A., Zou B. (2018). Pressure-induced emission of cesium lead halide perovskite nanocrystals. Nat. Commun..

[cit16] Wang L., Yao P., Wang F., Li S., Chen Y., Xia T., Guo E., Wang K., Zou B., Guo H. (2020). Pressure-Induced Structural Evolution and Bandgap Optimization of Lead-Free Halide Double Perovskite (NH_4_)_2_SeBr_6_. Adv. Sci..

[cit17] Ji F., Klarbring J., Wang F., Ning W., Wang L., Yin C., Figueroa J. S. M., Christensen C. K., Etter M., Ederth T., Sun L. (2020). Lead-Free Halide Double Perovskite Cs_2_AgBiBr_6_ with Decreased Band Gap. Angew. Chem..

[cit18] Clark S. J., Segall M. D., Pickard C. J., Hasnip P. J., Probert M. I., Refson K., Payne M. C. (2005). First principles methods using CASTEP. Z. Kristallogr..

[cit19] Hadi M. A., Dahlqvist M., Christopoulos S. R., Naqib S. H., Chroneos A., Islam A. K. M. A. (2020). Chemically stable new MAX phase V_2_SnC: a damage and radiation tolerant TBC material. RSC Adv..

[cit20] Perdew J. P., Burke K., Ernzerhof M. (1996). Generalized gradient approximation made simple. Phys. Rev. Lett..

[cit21] Vanderbilt D. (1990). Soft self-consistent pseudopotentials in a generalized eigenvalue formalism. Phys. Rev. B.

[cit22] Islam M. N., Hadi M. A., Podder J. (2019). Influence of Ni doping in a lead-halide and a lead-free halide perovskite for optoelectronic applications. AIP Adv..

[cit23] Roknuzzaman M., Zhang C., Ostrikov K. K., Du A., Wang H., Wang L., Tesfamichael T. (2019). Electronic and optical properties of lead-free hybrid double perovskites for photovoltaic and optoelectronic applications. Sci. Rep..

[cit24] Kumar N. R., Radhakrishnan R. (2018). Electronic, optical and mechanical properties of lead-free halide double perovskites using first-principles density functional theory. Mater. Lett..

[cit25] Greul E., Petrus M. L., Binek A., Docampo P., Bein T. (2017). Highly stable, phase pure Cs 2 AgBiBr_6_ double perovskite thin films for optoelectronic applications. J. Mater. Chem. A.

[cit26] Fischer T. H., Almlof J. (1992). General methods for geometry and wave function optimization. J. Phys. Chem..

[cit27] Hadi M. A., Roknuzzaman M., Nasir M. T., Monira U., Naqib S. H., Chroneos A., Islam A. K. M. A., Alarco J. A., Ostrikov K. K. (2021). Effects of Al substitution by Si in Ti 3 AlC 2 nanolaminate. Sci. Rep..

[cit28] Murnaghan F. D. , Finite deformation of an elastic solid, Wiley, 1951

[cit29] Bartel C. J., Sutton C., Goldsmith B. R., Ouyang R., Musgrave C. B., Ghiringhelli L. M., Scheffler M. (2019). New tolerance factor to predict the stability of perovskite oxides and halides. Sci. Adv..

[cit30] Hoke E. T., Slotcavage D. J., Dohner E. R., Bowring A. R., Karunadasa H. I., McGehee M. D. (2015). Reversible photo-induced trap formation in mixed-halide hybrid perovskites for photovoltaics. Chem. Sci..

[cit31] Yang J., Zhang P., Wei S. H. (2017). Band Structure Engineering of Cs_2_AgBiBr_6_ Perovskite through Order–Disordered Transition: A First-Principle Study. J. Phys. Chem. Lett..

[cit32] McClure E. T., Ball M. R., Windl W., Woodward P. M. (2016). Cs_2_AgBiX_6_ (X= Br, Cl): new visible light absorbing, lead-free halide perovskite semiconductors. Chem. Mater..

[cit33] Slavney A. H., Hu T., Lindenberg A. M., Karunadasa H. I. (2016). A bismuth-halide double perovskite with long carrier recombination lifetime for photovoltaic applications. J. Am. Chem. Soc..

[cit34] Lan C., Zhao S., Luo J., Fan P. (2018). First-principles study of anion diffusion in lead-free halide double perovskites. Phys. Chem. Chem. Phys..

[cit35] Zhou J., Xia Z., Molokeev M. S., Zhang X., Peng D., Liu Q. (2017). Composition design, optical gap and stability investigations of lead-free halide double perovskite Cs_2_AgInCl_6_. J. Mater. Chem. A.

[cit36] Luo B., Wang X., Lic E. T. G., Li L. (2015). Electronic structure, optical and dielectric properties of BaTiO_3_/CaTiO_3_/SrTiO_3_ ferroelectric superlattices from first-principles calculations. J. Mater. Chem. C.

[cit37] Den Toonder J. M. J., Van Dommelen J. A. W., Baaijens F. P. T. (1999). The relation between single crystal elasticity and the effective elastic behaviour of polycrystalline materials: theory, measurement and computation. Modell. Simul. Mater. Sci. Eng..

[cit38] Pettifor D. G. (1992). Theoretical predictions of structure and related properties of intermetallics. Mater. Sci. Technol..

[cit39] Hadi M. A., Islam M. N., Babu M. H. (2019). Cubic Perovskite Pb(Mg_1/3_Nb_2/3_)O_3_: A Damage Tolerant, Machinable, and Thermal barrier coating material. Z. Naturforsch., A: Phys. Sci..

[cit40] Dong L., Sun S., Deng Z., Li W., Wei F., Qi Y., Li Y., Li X., Lu P., Ramamurty U. (2018). Elastic properties and thermal expansion of lead-free halide double perovskite Cs_2_AgBiBr_6_. Comput. Mater. Sci..

[cit41] Born M. (1940). On the stability of crystal lattices. I. Proc. Cambridge Philos. Soc..

[cit42] Gaillac R., Pullumbi P., Coudert F. X. (2016). ELATE: an open-source online application for analysis and visualization of elastic tensors. J. Phys.: Condens. Matter.

[cit43] Chini M. K., Srinivasan S. G., Tailor N. K., Salahub D., Satapathi S. (2020). Lead-free, stable mixed halide double perovskites Cs_2_AgBiBr_6_ and Cs_2_AgBiBr_6−*x*_Cl_*x*_ -A detailed theoretical and experimental study. Chem. Phys..

[cit44] Roknuzzaman M., Ostrikov K. K., Wang H., Du A., Tesfamichael T. (2017). Towards lead-free perovskite photovoltaics and optoelectronics by *ab initio* simulations. Sci. Rep..

[cit45] Ayatullahah H., Murtaza G., Muhammad S., Naeem S., Khalid M. N., Manzar A. (2013). Physical properties of CsSnM_3_ (M = Cl, Br, I): A first principle study. Acta Phys. Pol., A.

[cit46] Liu X., Xie B., Duan C., Wang Z., Fan B., Zhang K., Lin B., Colberts F. J., Ma W., Janssen R. A., Huang F. (2018). A high dielectric constant non-fullerene acceptor for efficient bulk-heterojunction organic solar cells. J. Mater. Chem. A.

